# Novel vancomycin resistance gene cluster in *Enterococcus faecium* ST1486, Belgium, June 2021

**DOI:** 10.2807/1560-7917.ES.2021.26.36.2100767

**Published:** 2021-09-09

**Authors:** Basil Britto Xavier, Jasmine Coppens, Sien De Koster, Sahaya Glingston Rajakani, Sam Van Goethem, Samy Mzougui, Ahalieyah Anantharajah, Christine Lammens, Katherine Loens, Youri Glupczynski, Herman Goossens, Veerle Matheeussen

**Affiliations:** 1 Laboratory of Medical Microbiology, Universiteit Antwerpen, Wilrijk, Belgium; 2 Vaccine and Infectious Disease Institute, Universiteit Antwerpen, Wilrijk, Belgium; 3 Laboratory of Clinical Microbiology, Antwerp University Hospital, Edegem, Belgium; 4 Laboratory of Clinical Microbiology, Cliniques Universitaires Saint-Luc-UCLouvain, Brussels, Belgium; 5 Belgian National Reference Centre for Enterococci, Antwerp University Hospital, Edegem, Belgium

**Keywords:** **:***E. faecium*, ST1486, novel van gene cluster, *vanP*, CC17

## Abstract

We identified a novel *van* gene cluster in a clinical *Enterococcus faecium* isolate with vancomycin minimum inhibitory concentration (MIC) of 4 µg/mL. The ligase gene, *vanP*, was part of a *van* operon cluster of 4,589 bp on a putative novel integrative conjugative element located in a ca 98 kb genomic region presumed to be acquired by horizontal gene transfer from *Clostridium* *scidens* and *Roseburia* sp. 499. Screening for *van* genes in *E. faecium* strains with borderline susceptibility to vancomycin is important.

Enterococci are part of the regular intestinal microbiota in humans and animals. In the past two decades, *E. faecium* has rapidly evolved as a worldwide nosocomial pathogen by successfully adapting to the hospital environment [1,2]. The World Health Organization (WHO) has described vancomycin-resistant *E. faecium* (VRE) as a high-priority pathogen with urgent need of new treatments [3]. In the present study, we present a case infected with a borderline vancomycin-susceptible *E. faecium* #21122516 with a minimum inhibitory concentration (MIC) of 4 µg/mL, which grew on a chromogenic VRE-selective agar after a 48 h incubation but was PCR-negative for all known *van* genes.

## Case report

In January, 2021, a woman in her 50s was admitted for cancer surgery to one hospital in Belgium. Her postoperative status in the intensive care unit (ICU) was complicated by the occurrence of ventilator-associated pneumonia and bacteraemia for which she received multiple courses of broad-spectrum antibiotics (ceftazidime, meropenem, piperacillin-tazobactam, vancomycin, ciprofloxacin) spanning over a 12-week period. The patient recovered and was transferred after 3 months to a physical rehabilitation unit. Upon admission to the ICU, multiple screening swabs (rectal, upper and lower respiratory tract) taken to detect extended spectrum beta-lactamases, carbapenemase-producing Enterobacteriacae and methicillin-resistant *Staphylococcus aureus* were negative. However, screening for VRE was not done. After the end of antibiotic therapy (early March 2021), one routine urine specimen yielded a mixed growth of *bla*_NDM-1_ carbapenemase-producing *Enterobacter cloacae* and of *E. faecium* #21122516, which on BD Phoenix PMIC Panels (New Jersey, United States (US)) was borderline susceptible to vancomycin (MIC 4 µg/mL) and susceptible to teicoplanin (MIC < 1 µg/mL). Because this *E. faecium* strain also grew on a chromogenic selective agar (Chrom ID VRE, bioMérieux, Marcy-l'Étoile, France) after 48 h, the isolate was sent as a putative VRE to the Belgian National Reference centre for Enterococci (UZA, Antwerpen, Belgium) for genetic confirmation.

## Identification of a novel *van* gene cluster in a clinical *Enterococcus faecium* isolate not harbouring any known *van* genes

**Figure 1:**
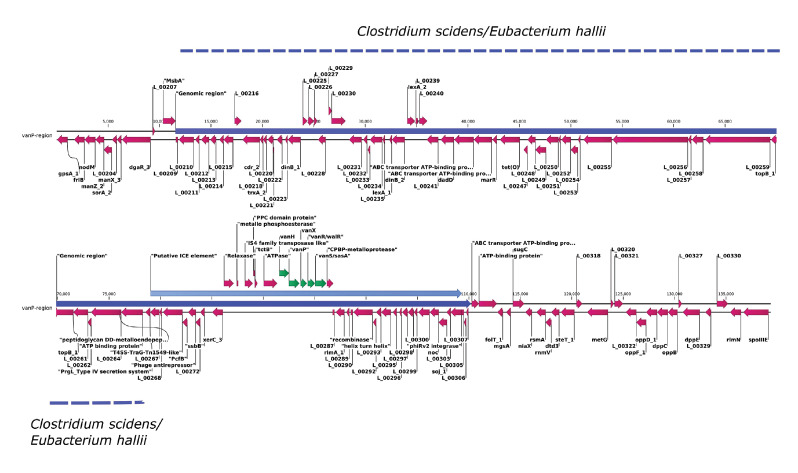
Genetic organisation of a novel *van* gene cluster in a putative ICE element, clinical isolate of *Enterococcus faecium*, Belgium, June 2021

Genomic DNA was isolated by use of MagAttract HMW DNA kit (Qiagen, Hilden, Germany) library preparation (Nextera XT), sequenced by MiSeq (2 × 250 bp, Illumina, San Diego, US). The sequencing data of this study are deposited under the BioProject ID PRJNA741920. Data analysis was done using BacPipe v.2.6.1 [4]. BacPipe results showed the absence of *van* genes after screening against Resfinder and CARD databases. However, Resfams gave hits to putative glycopeptide resistance genes [5]. The hits containing scaffolds were further analysed using CLC Genomics workbench v.20.1 (clcbio, Aarhus, Denmark) and identified as follows: *vanH-vanP-vanX-vanR/walR-vanSG/sasA* with 4,586 bp (Figure 1). BLAST analysis (blastn) of this whole operon gave no hits to any of the organisms in the NCBI non-redundant protein database on 23 July 2021. However, in a BLASTX-based search, the D-alanine-D-alanine ligase domain (WP_075721811) had the highest identity (71.89%) to *Roseburia* sp. 499.

ICE: integrative conjugative element.

Green: *van* gene cluster; light blue: putative ICE element; dotted lines: indicating genomic region shown similarity to *Clostridium scidens*; dark blue: acquired genomic region.

## Emergence of resistance to vancomycin and teicoplanin following genetic modifications in the ligase gene in serial passaging

By microdilution method, *E. faecium* #21122516 (vancomycin (MIC 4 µg/mL) and teicoplanin (MIC of ≤ 1 µg/mL)) was serially passaged three times in the presence of sub-inhibitory concentrations of vancomycin (2 µg/mL) along with a vancomycin-susceptible (MIC of 1 µg/mL) control strain *E. faecium* #21232802 on a Sensititre Gram-positive EUSTAPF plate (ThermoFisher Scientific, Cleveland, US). 

While the vancomycin MIC of the control strain *E. faecium* #21122516 remained unchanged, resistance emerged for the *E. faecium* #21232802 after the first passage (one-fold increase from MIC 4 to 8 µg/mL for vancomycin) while the teicoplanin MIC rose from 0.5 to 2 µg/mL. After a second passage, MIC values rose to > 256 µg/mL for vancomycin and 4 µg/mL for teicoplanin. Finally, clones after the third passage showed a marked increase in the MIC to both vancomycin (> 256 µg/ml) and teicoplanin (8 µg/mL). The series of three passages was done three times with the same results.

**Figure 2:**
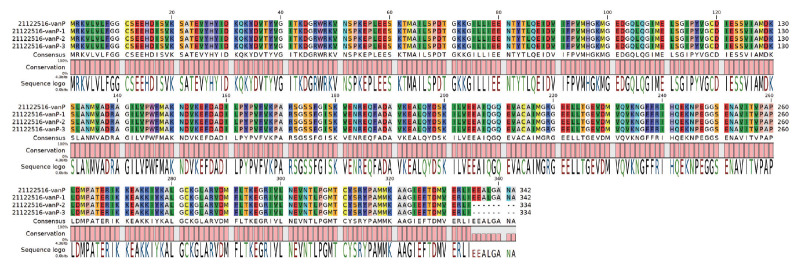
VanP ligase protein sequence compared parental strain with passaged strains, clinical *Enterococcus faecium* isolate, Belgium, June 2021

In order to confirm the genetic modifications, whole genome sequencing of in vitro clones from the second and third passages showed a non-synonymous mutation (G to T) in the ligase gene (*vanP*) (changing Glu 335 to a stop codon) compared with the original strain and with clones from the first passage. This premature stop codon led to a ligase open reading frame 24 nt shorter than that in the parental and once passaged strains (Figure 2).

Acquired vancomycin and teicoplanin resistance of *E. faecium* #21122516 clones was shown to be stable in vitro after five consecutive passages after the appearance of resistance in an antibiotic-free medium.

## Acquisition of a large genomic region including a novel *van* gene cluster on a putative ICE element

We screened the *van* gene cluster-containing scaffold for an integrative *conjugative element* (ICE) using ICEberg 2.0 (http://db-mml.sjtu.edu.cn/ICEberg/) [6]. It predicted the presence of a putative ICE element ca 30 kb in size, harbouring *van* genes. This putative ICE element was part of a large genomic region (size ca 98.6 kb) acquired by *E. faecium* #21122516 and all the components such as type 4 secretion system of Gram-positive ICE elements were identified and annotated (data not shown). In order to determine the insertion site, we performed a comparative genome analysis between *E. faecium* #21122516 and *E. faecium* of non-clinical origin NRRL B-2354 (GenBank accession number CP004063.1). Acquisition had occurred in the insertion site sequence (CAC AAT) between 2,251,669 and 2,252,800 bp, in the coding sequence of *msbA* for a lipid A export ATP-binding protein. The whole 98.6 kb region showed similarity to *Clostridium scidens* (66% query coverage; 99.26% identity) and to *Eubacterium hallii* (68% query coverage: 99.57% identity). On the other hand, we did not find any similarities to *E. faecium* or any enterococcal genomes when we searched against the NCBI nr database (Figure 1).

## Molecular analysis of D-Ala-D-X ligases from *Enterococcus faecium* #21122516

**Figure 3:**
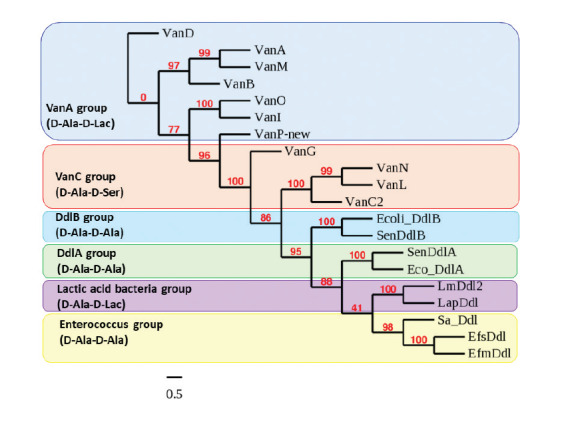
Phylogenetic relatedness of D-Ala-D-X ligases from different bacterial species

In order to assess the new gene D-Ala-D-X ligase group, we did a phylogenetic analysis of the protein sequences of all known ligase genes taken from GenBank. We further divided the D-Ala-D-X ligases into six major groups based on their phylogenetic relationships. Three were D-Ala-D-Ala ligases, two were D-Ala-D-Lac and one was a D-Ala-D-Ser ligase. The new ligase gene *vanP* was distantly related to other D-Ala-D-Lac sequences, but clustered within the VanA group (Figure 3).

This figure is adapted from Djalal Meziane-Cherif et al. [17]. Bootstrapping analysis of 1,000 replicates was performed using MEGA-X [18] to assess the evolutionary relationship between ligases. Branch lengths represent the number of substitutions per site. Bootstrap values are indicated in the nodes.

## Genetic characterisation of *Enterococcus faecium #*21122516

The *E. faecium #*21122516 strain belongs to sequence type ST1486 of the CC17 complex. In line with the characteristics of a hospital-adapted lineage, this strain harboured multiple resistance genes for trimethoprim (*dfrG*), tetracycline (*tetO*), aminoglycoside (*aac(6’)-Ii, ant(6’)-Ia*), *aph (3’)-III,* macrolide (*msr(C)* and virulence factors involved in adhesion to collagen and to adhesive matrix molecules like *acm* (a collagen adhesin precursor), *sgrA,* a cell well-anchored protein that stimulates surface adhesion, *ecbA* collagen-binding protein A and the *esp* enterococcal surface protein, which promote biofilm formation. The strain harbours rep14a, rep2 and repUS15 plasmid replicons. We detected eight incomplete phages using the PHASTER online tool [7], indicating that the genome is prone to acquiring new genes.

**Figure 4:**
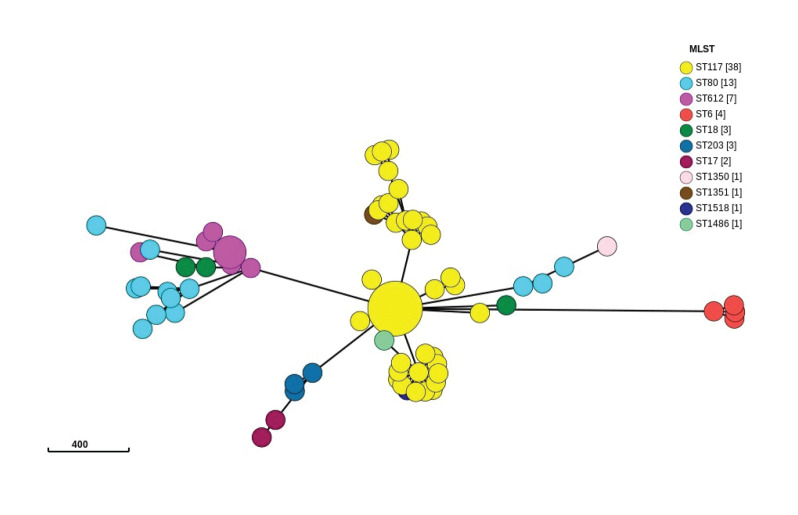
Minimum spanning tree comparing core genomic allelic profiles in association with VRE clinical strains isolated in the past 5 years, Belgium 2017–2021

MLST: multilocus sequence typing; VRE: vancomycin-resistant *Enterococcus faecium*.

We looked into the progenitor of *E. faecium* ST1486 harbouring the *van* gene cluster. We performed the gene-by-gene approach to determine the core genome [8] multilocus sequence typing (MLST) and whole genome MLST allelic loci distance using ChewBBACA [9] and visualised it through Grapetree [10] of the strains sequenced in the past 5 years (Figure 4). A total of 74 *E. faecium* clinical strains from CC17 complex were randomly selected among the isolates to the reference centre in the period from 2017 to 2021 (Figure 4).

Isolates are displayed as circles. Numbers in brackets refer to the number of isolates. The distance between S1486 and ST117 was 79 allelic loci, and both sequence types belonged to the CC17 cluster.

## PCR screening for the new *vanP* gene

The new *vanP* gene can be detected by a standard PCR-based screening assay using primers VANP-IF 5’ ATGCAGACATTCTTCCTTATCC 3’ and VANP-IR 5’ GTAACTCCTCTCCTCTTCCC 3’, and the following cycling conditions: 95 °C × 3 min, 25 cycles of 98 °C × 20 s, 55 °C × 15 s, 68 °C × 30 s and one cycle of 72 °C × 2 min.

## Ethical statement

Approval by an ethics committee was not required for this study because the informed consent from the patient was obtained before investigation. All data were anonymised to protect patient privacy and confidentiality.

## Discussion

We found a newly acquired glycopeptide resistance *van* gene cluster(*vanP*) in a clinical strain of *E. faecium* which had occurred as a sporadic isolate in a hospitalised patient following multiple courses of antimicrobial therapy. Despite the fact that molecular screening for all known *van* genes yielded a negative result, we found that resistance to vancomycin and to teicoplanin could be acquired easily and rapidly following exposure to subinhibitory vancomycin concentrations. We could also show that a non-synonymous mutation in the ligase gene created a premature stop codon in the passaged clones, associated with an increase in the vancomycin and teicoplanin MIC. However, actual functional modifications are yet to be explored.

An increase in VRE isolates has been attributed to clonal expansion following gene acquisition [11]. We propose that ST1486 has arisen independently through horizontal gene transfer in hospitals [12]. *Roseburia* is a Gram-positive intestinal bacterium, and member of Clostridiales, obligate anaerobes that are motile and reported to be difficult to culture and highly susceptible to antimicrobial agents, but homologous *vanG*-like elements have been reported in *Roseburia* spp. isolates from the human intestine [13]. As for other types of enterococci, the acquisition and carriage of antibiotic resistance genes is one possible explanation for why *E. faecium* could spread in a hospital setting so quickly [14].

Of the 12 identified *van* genes, nine have been observed in clinical isolates (*vanA*, *B, C, D, G, L, M, N*) [2,15,16]. It is acknowledged that the predominance of *vanA* and *vanB* clusters in major outbreaks is partly due to these clusters being part of successful mobile genetic elements that can disseminate between successful clones. However, selection bias may also contribute to this dominance since in most studies, screening is only performed for the *vanA* and *vanB* genes [2].

### Conclusion

We found a novel *van* gene cluster, *vanP*, which was associated with a low level of expression of resistance to vancomycin in a patient clinical isolate of *E. faecium* but caused high-level vancomycin resistance and medium-level teicoplanin resistance after exposure to sub-inhibitory concentrations of vancomycin. We hypothesise that this novel *van* gene cluster was most probably acquired during co-colonisation of *Clostridium* *scidens* and *Roseburia* sp. 499 via horizontal gene transfer under selective pressure by antibiotics. These data suggest that pathogenic enterococci can acquire new vancomycin resistance genes from non-pathogenic species, which can be missed when performing routine screening for vancomycin resistance that is limited to *vanA* and *vanB* resistance genes. There is a risk that if they remain unnoticed, some of these strains could proliferate in the hospital setting and pose a threat in the future.
